# Embolization of infective endocarditis vegetation causes intracranial hemorrhage with hemorrhagic transformation after ischemic stroke

**DOI:** 10.21542/gcsp.2024.2

**Published:** 2024-01-03

**Authors:** Averina O. Aslani, Zahran Haryawan, Tania A. Sabrawi, Robin H. Wibowo, Aprilianasary U. Dewi

**Affiliations:** 1 Sumedang Regional General Hospital, Sumedang, West Java, Indonesia; 2 Ciamis Regional General Hospital, Ciamis, West Java, Indonesia

## Abstract

Background: Infective endocarditis (IE) is a rare, but potentially fatal, infectious disease. One of the common complications of IE is the embolization of endocardial vegetation with subsequent intracerebral artery obstruction that causes acute ischemic stroke. Herein, we present a case report of a patient presenting with a neurological manifestation that turned out to be a complication of IE.

Case Illustration: We present the case of a patient with a chief complaint of left-sided hemiplegia. Blood test results revealed signs of infection. Computed tomography (CT) of the head revealed extensive infarction in the right lobe and subarachnoid hemorrhage. Echocardiography revealed vegetation on the aortic valve, suggesting that IE was the source of embolization. The patient was treated with high-dose ampicillin and gentamicin, supportive stroke therapy, and physical rehabilitation.

Conclusion: IE can be considered one of the causes of acute ischemic or hemorrhagic stroke. Ruling out other common causes of stroke and noticing signs of infection and vascular phenomena helps define the diagnosis. Echocardiography helps identify valvular vegetation to support the diagnosis. Treatment consists of high-dose penicillin and supportive therapy for stroke.

## Background

Infective endocarditis (IE) is a rare, but potentially fatal, infectious disease. According to reports, the mortality rate of IE can reach 20%, with the morbidity rate ranging from 1.5 to 11.6/100,000^1^. Typically, patients with previously identified heart disease or a history of open heart surgery who experience fever, widespread weakness, weight loss, and/or signs of a systemic embolism are suspected of having IE^2^. However, a recent investigation of 328 consecutive Korean patients with IE in a tertiary referral hospital found that 76% (249 patients) at the time of hospitalization for IE had no prior medical history of an underlying heart condition^3^. IE patients are frequently admitted due to complications, the most common of which are congestive heart failure caused by valvular lesions, septic shock, especially when *Staphylococcus aureus* is involved, and neurological complications^4^.

One of the common complications of IE is the embolization of endocardial vegetation with subsequent intracerebral artery obstruction that causes acute ischemic stroke^5^. Stroke is among the most significant risk factors for death in IE patients, accounting for 55–58% of the mortality rate^6^. In individuals with infective endocarditis, hemorrhagic stroke occurs in approximately 5% of cases^7^. The most common cause of these bleedings is ruptured mycotic aneurysms^8^. Ruptured septic erosion of the arterial wall may also cause hemorrhagic stroke^9^. Ischemic injury can also lead to an inflammation process that alters the integrity of blood vessels and triggers a hemorrhagic event^10^. Here we present a case report of a patient who presented with a neurological manifestation that turned out to be the complication of IE. We hope that by analyzing this case and evaluating recent literature, this paper will help bring attention to IE diagnosis, treatment, and associated neurovascular consequences.

## Case illustration

A 31-year-old male presented with a chief complaint of left hemiparesis and loss of consciousness five days prior to admission. Two days before admission, the patient regained consciousness but still had left hemiparesis. These complaints were accompanied by weight loss, coughing, fever, and poor dental hygiene.

Physical examination revealed tachycardia and oral candidiasis. Electrocardiography indicated sinus tachycardia. Neurological examination revealed left lateralization, pathological reflex on the left side, nuchal rigidity, and a score of 0 (zero) for motor function of the upper and lower left extremities.

Laboratory investigation of blood taken on the same day revealed a low haemogoblin of 9.6 g/dl (reference value of 14–17.5 g/dl), decreased erythrocyte count of 3.30 million/µl (reference value of 4.5–5.9 million/µl), decreased hematocrit of 27.6% (reference value of 40–52%), and increased leukocyte count of 26,700/mm^3^ (reference value of 4500–10,000/mm^3^). Blood workup showed a normal erythrocyte index, with a differential count of leukocytes showing hypersegmentation. Examination of the ureum and creatinine levels revealed normal kidney function. Electrolyte examination revealed sodium level of 122 mmol/l (reference value of 135–148 mmol/l) and calcium level of 7.93 mmol/l (reference value of 8.1–10.4 mmol/l). An X-ray photo of the thorax showed no signs of infection or active tuberculosis.

**Figure 1.**
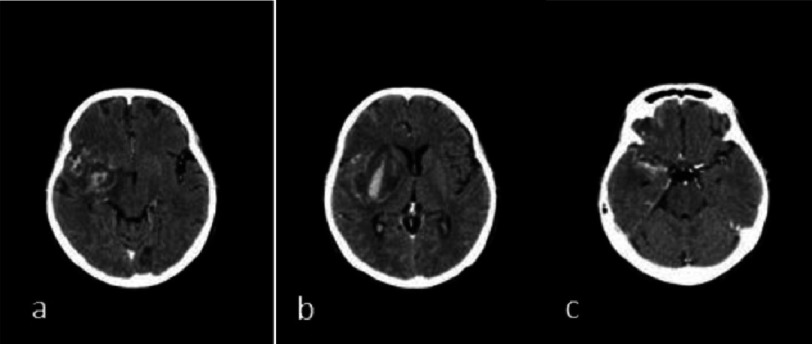
(a) & (b) hemorrhagic transformation following cerebral ischemia, (c) subarachnoid hemorrhage.

After five days, the patient underwent a head CT scan examination. There was extensive infarction accompanied by hemorrhagic transformation in the cortical-subcortical area of the right frontotemporoparietal lobe and right basal ganglia, which caused a 4 mm midline shift towards the left side. The CT scan also revealed subarachnoid hemorrhage that occupied some parts of the sulci corticalis and the right Sylvian fissure (Figure 1).

**Figure 2.**
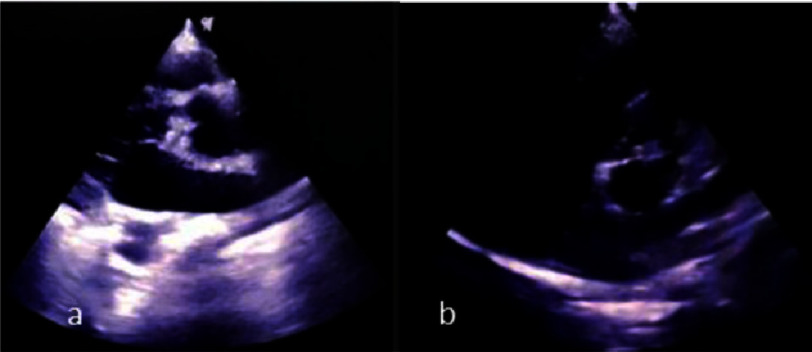
Initial TTE showing vegetation at aortic valve (RCC, LCC) ±22–24 mm. (a) PLAX view (b) PSAX view.

No intracranial space-occupying lesions were observed. The patient was then referred to a cardiologist for echocardiography examination to identify the possible source of embolization that caused the infarction. Transthoracic echocardiography (TTE) revealed moderate to severe aortic regurgitation, dilated left ventricle, reduced ejection fraction of 47%, and possible vegetation at the aortic valve at the left coronary cusp (LCC) and right coronary cusp (RCC), suggesting the diagnosis of IE (Figure 2).

**Figure 3.**
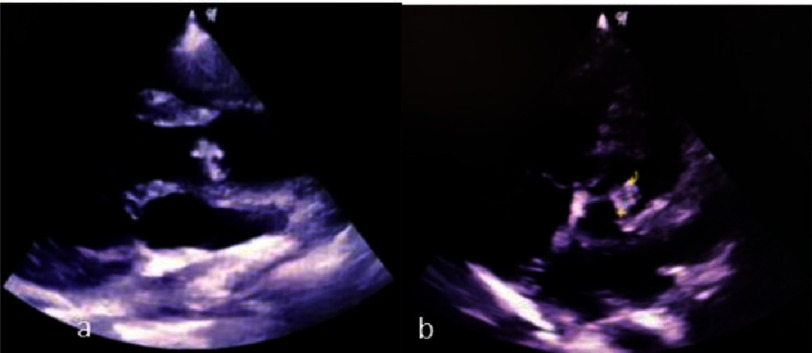
TTE after treatment showing vegetation at RCC & LCC ±13–14 mm. (a) PLAX view (b) PSAX view.

The patient was treated with NaCl 0.9% fluid rehydration, citicoline (2  × 500 mg IV), omeprazole (1  × 40 mg IV), potassium chloride (1  × 600 mg PO), and 500 mg paracetamol injection if there was any fever. After the cardiologist consultation, the patient was administered ampicillin sulbactam (4  × 3 g IV), gentamicin (1  × 160 mg IV), ramipril (2 × 2.5 mg PO), and bisoprolol (1 × 2.5 mg PO). The patient was eventually discharged after significant improvement in clinical and laboratory findings, and weekly echocardiography. The subsequent echocardiography revealed the reduction of vegetation size (Figure 3). The patient was also consulted to the physical rehabilitation department to improve his range of motion.

## Discussion

A wide range of arterial injuries, from acute, pyogenic necrosis to sizable, aseptic aneurysms that may burst weeks to months after bacteriologic infection, result in stroke complicating infective endocarditis^9^. This complication is primarily caused by the embolism of vegetation in the cerebral circulation; the lesion may be suppurative, inflammatory, or merely ischemic depending on the type of embolism (septic or not)^11^. Acute erosive arteritis with rupture can be caused by septic emboli during uncontrolled infection, particularly with virulent pathogens, as we believe in our case.

The transformation from ischemic to hemorrhagic stroke might also be caused by inflammation. Acute cerebral ischemia severely damages capillary cells, increases vascular permeability, and allows extravasation of blood into brain parenchyma. Oxidative stress and reperfusion injury cause damage to blood vessels through a variety of mechanisms, including inflammation, leukocyte infiltration, vascular activation, and extracellular proteolysis. As a result, the basal lamina and tight endothelial connections are damaged. This leads to subsequent hemorrhagic process^10^.

Endocarditis is typically considered a potential cause of stroke when other more common causes of stroke have been ruled out and the patient exhibits symptoms of fever, sepsis, or repeated embolic episodes. There is no test that can quickly and accurately demonstrate endocarditis, apart from echocardiography, which is a user-dependent test and thus prone to human error^12^. As in our case, the IE was taken into the consideration after the CT scan showed no sign of intracranial infection, no history or physical sign of symptoms that led to thrombotic source, and the blood workup showed sign of active infection.

The classic Duke criteria are commonly used; however, they still have flaws. First, specific organisms are labeled as major criteria, making the possibility of culture-negative IE seem remote. This can make it more difficult to diagnose IE because not all microbiology laboratories can run tests for uncommon agents that are increasingly being mentioned in connection with IE (such as *Tropheryma whipplei*). Second, cardiac ultrasonography is a method open to individual interpretation and occasionally erroneous (with obese or poorly compliant patients). Since not all infectious organisms cause high-grade fevers, and because vascular conditions such as Janeway lesions and some immunologic conditions such as Osler nodes are uncommon (10% of cases), most clinicians are not familiar with them, making them frequently inapplicable. These uncommon clinical manifestations were not observed in the present case. Finally, embolic phenomena, particularly in the event of stroke, should certainly play a bigger part in the diagnosis of IE because they may have a greater impact on the patient’s outcome and need the use of a particular antibiotic^13^. Further studies are needed to define more applicable diagnostic criteria for IE.

The patient in this case showed improvement when the antibiotics were administered, while days before there was no improvement, as the diagnosis of IE was not yet determined. As the patient was treated at a regional hospital in a remote area, the culture procedure could not be performed; thus, the patient was treated with broad-spectrum antibiotics.

While proven ruptured mycotic aneurysms are recorded in only approximately 1.7% (range 0.8–2.8%) of patients during the acute phase of infective endocarditis, hemorrhage occurs in approximately 5% of individuals. Owing to deadly initial hemorrhage or multiplicity of aneurysms, only approximately one-third of patients with ICH and burst mycotic aneurysms undergo surgical repair^14^. While our patient had vegetation identified in the aortic valve, some studies have revealed that cases of left mitral endocarditis have a higher incidence of cerebral emboli than cases of aortic endocarditis^15^. The extent of vegetation and presence of Staphylococcus are the two most important predictors. Studies using imaging (brain scan) or clinical methods to identify cerebral embolic events have demonstrated that the risk of an embolic event considerably increases when the size of the vegetation is >10 mm. Chronology also affects the embolic risk. Regardless of the size of the vegetation, it declines following the start of curative antibiotic therapy and weakens after two weeks^16^.

In addition to providing supportive stroke care, embolic risk should be reduced. Anticoagulation is the strategy for secondary prevention of thrombotic events, while antibiotic therapy remains the cornerstone of IE^17^. Despite encouraging outcomes in experimental research, there is insufficient evidence to warrant the initiation of anticoagulant medication in individuals with IE^16^. The unclear risk of rupture dampens the enthusiasm for identifying unruptured mycotic aneurysms. Many case studies attest to the fact that, after receiving antimicrobial medication, many unruptured mycotic aneurysms, particularly large and expanding ones, heal completely. It is unknown how common unruptured intracranial mycotic aneurysms are in people with endocarditis^18^. However, as the CT scan revealed a subarachnoid hemorrhage, anticoagulants were not administered to the patient.

### What have we learned?

Acute ischemic and hemorrhagic stroke are among the most common complications of IE. It is associated with increased morbidity and mortality. As it tends to occur in patients admitted with neurological manifestations, IE is often diagnosed only when other possibilities of stroke are ruled out. Echocardiography is routinely performed to detect the possible sources of embolism. The principles of therapy involve supporting therapy for neurological symptoms and eradicating pathogens using high-dose penicillin.

## Data availability

This study was not supported by any data.

## Consent

The patient was sufficiently anonymized, thus the institutional ethical committee did not require written informed consent.

## Disclosure

The case described in this manuscript was included in an abstract and displayed poster session titled “Ischaemic Stroke and Subarachnoid Haemorrhage as the Complication of infective Endocarditis in Young Male: a Case Report” that was presented at the “32nd Annual Scientific Meeting of Indonesian Heart Association” in Indonesia on May 25–28, 2023. To prevent self-plagiarism, significant changes were made to the abstract before it was included in this publication.

## Conflict of Interests

The authors declare that they have no conflicts of interest.

## Authors Contribution

**Conceptualization:** Averina O. Aslani, Zahran Haryawan, and Tania A. Sabrawi.

**Data curation:** Averina O. Aslani.

**Formal analysis:** Averina O. Aslani and Zahran Haryawan.

**Investigation:** Averina O. Aslani and Tania A. Sabrawi.

**Methodology:** Aprilianasary U. Dewi.

**Project administration:** Averina O. Aslani.

**Supervision:** Tania A. Sabrawi and Robin H. Wibowo.

**Validation:** Averina O. Aslani, Zahran Haryawan, Tania A. Sabrawi, Robin H. Wibowo, and Aprilianasary U. Dewi.

**Writing - original draft:** Averina O. Aslani and Zahran Haryawan.

**Writing - review & editing:** Averina O. Aslani and Zahran Haryawan.
